# Aggressive squamous cell carcinoma of the bladder associated with a history of large bladder stone – a case report

**DOI:** 10.1002/ccr3.1133

**Published:** 2017-08-22

**Authors:** Manoj Hilary Fernando, Umesh Jayarajah, Kasun Bandara Herath, M. Vipula Chandu de Silva, Serozsha Anura Sahadeva Goonewardena

**Affiliations:** ^1^ Department of Urology National Hospital of Sri Lanka Colombo Sri Lanka; ^2^ Department of Pathology Faculty of Medicine University of Colombo Sri Lanka

**Keywords:** Case report, large bladder stone, squamous cell bladder carcinoma

## Abstract

History of large bladder stones suggests a long‐standing chronic irritation of the bladder, a known risk factor for squamous cell carcinoma. Therefore, in such patients, we suggest random biopsies to detect presence of dysplasia or malignancy and a follow‐up cystoscopy for early detection of a possible tumor.

## Introduction

Squamous cell carcinoma of the bladder (SCCB) is uncommon in nonschistosoma endemic areas, accounting for 2.7% of bladder cancers in the developed world 1. A study from Sri Lanka showed that about 2.9% of bladder cancer were squamous cell carcinoma 2. Chronic bladder irritation with bladder calculi is a known predominant risk factor for SCCB. We present a case of a 57‐year‐old male patient presenting with muscle invasive SCCB with a history of large bladder calculus and recurrent stone formation.

## Case Presentation

**Figure 1:**
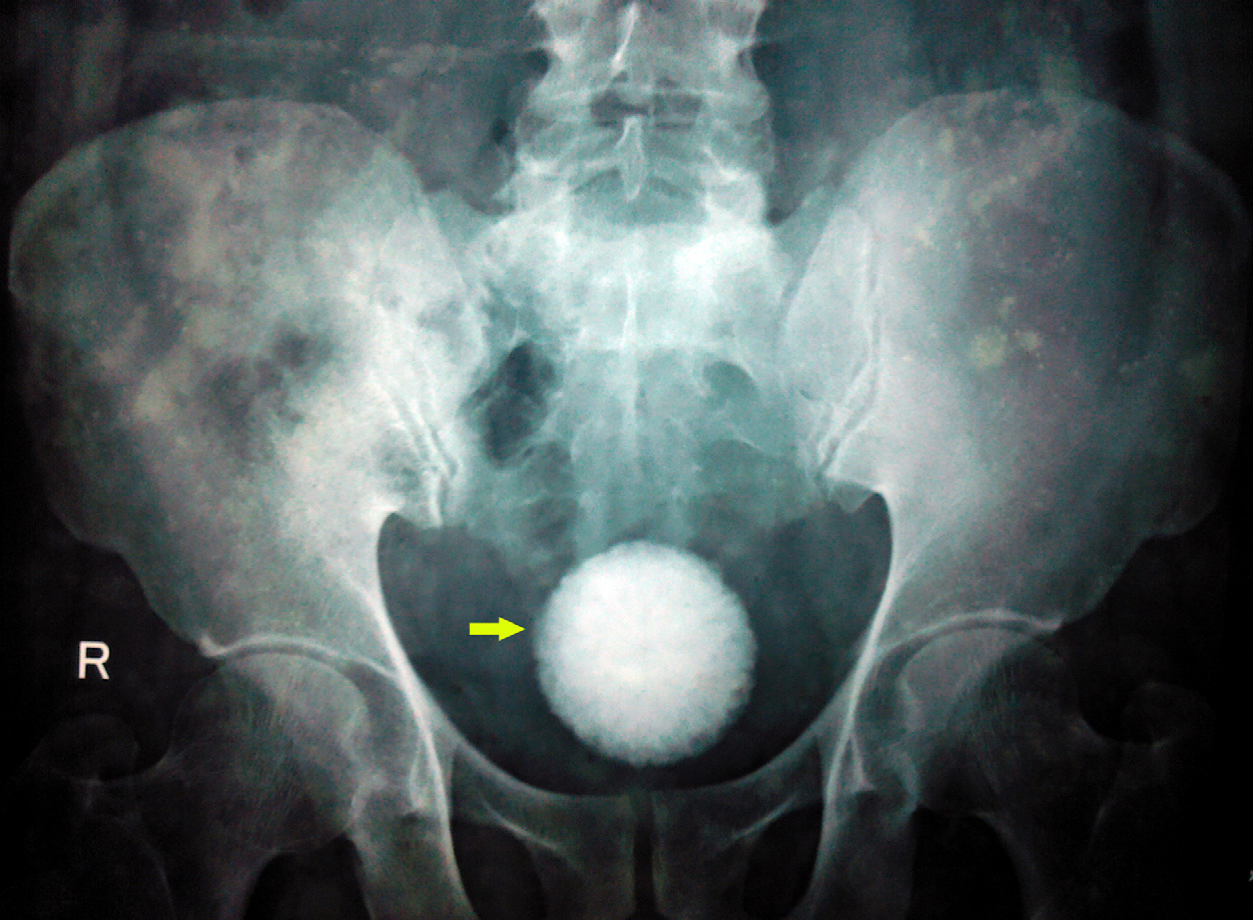
X‐ray KUB (3 years prior to presentation) showing a large bladder stone 5.5 × 5.6 cm.

A 57‐year‐old man with a history of open vesicolithotomy for a large bladder stone (size 5.5 × 5.6‐cm; Fig. 1) 3 years back, presented with progressive dysuria, intermittent flow and need to strain to pass urine for 3 months. He also had progressive worsening day time frequency of 10 times and nocturia of four times. Furthermore, he complained of episodes of intermittent complete hematuria over the past 3 weeks. He had type II diabetes mellitus which was well controlled. He had a history of smoking (five pack years) 20 years ago.

The general physical examination was unremarkable, and abdominal examination did not reveal any tenderness or a palpable bladder. The digital rectal examination revealed a firm, small prostate of 10–15 g which was clinically benign.

**Figure 2:**
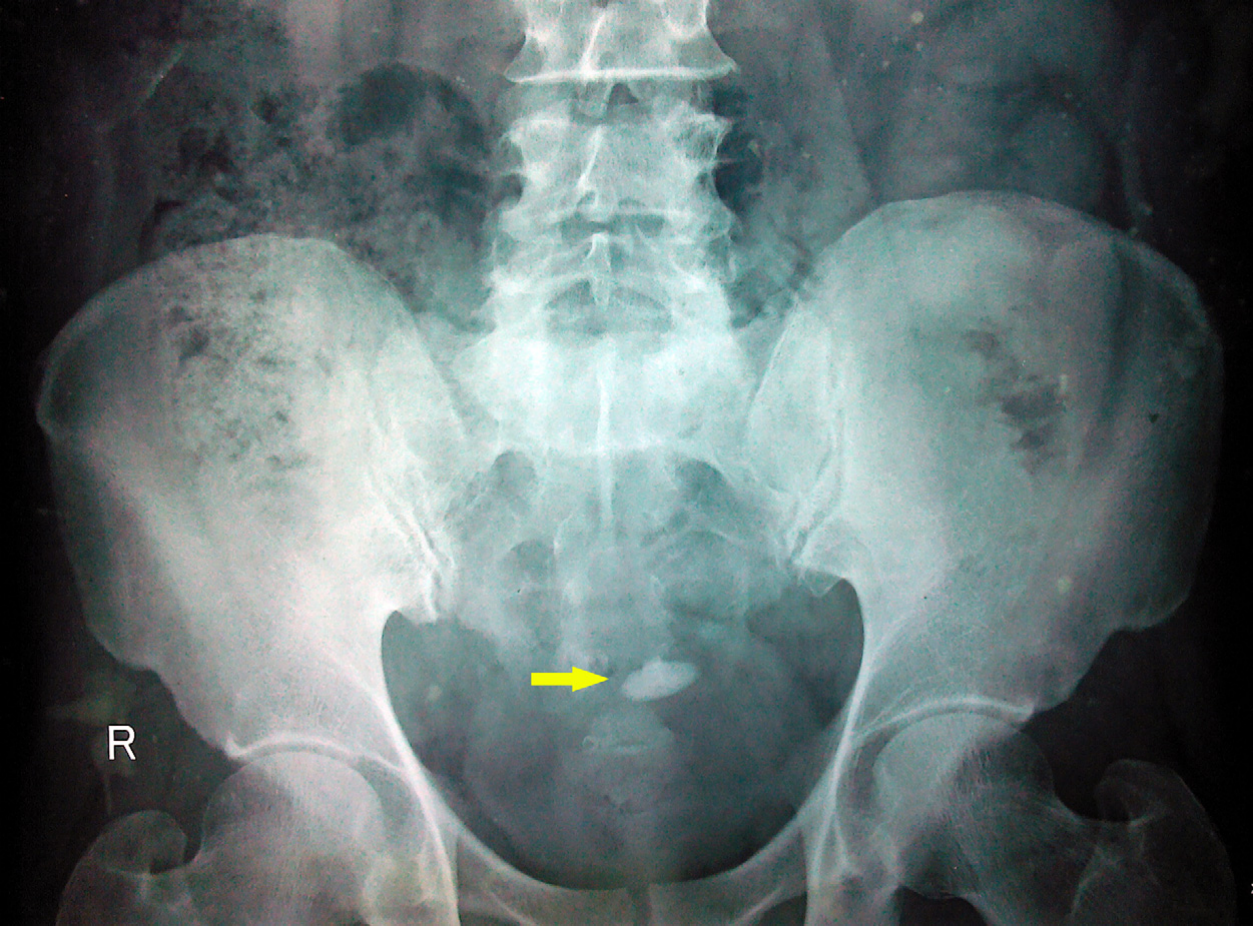
X‐ray KUB (at presentation) showed a 2.2 × 1.8 mm elongated bladder stone.

**Figure 3:**
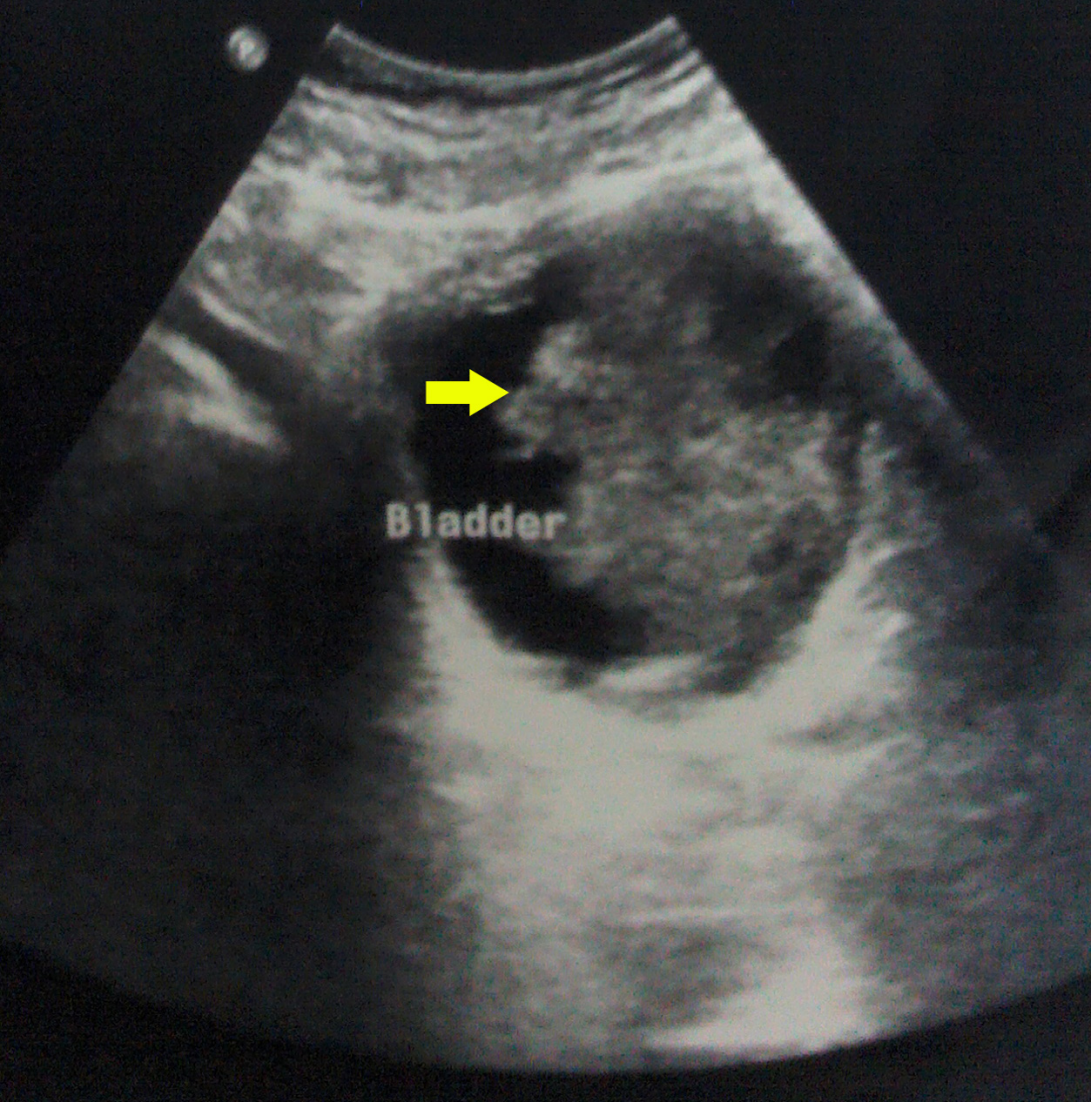
Ultrasonography showing a large echogenic irregular mass lesion with internal vascularity in the left lateral wall of the bladder projecting into the lumen.

His urine analysis showed 50–100 pus cells and 25–30 red cells per high power field. Urine culture yielded no significant growth. Uroflowmetry had a bell shaped curve with a maximum flow rate of 22 mL/sec for a voided urine volume of 176 mL. His X‐ray KUB (Kidney, ureters and bladder) showed a 2.2 × 1.8 cm elongated bladder stone (Fig. 2). Ultrasonography revealed a large echogenic irregular mass lesion with internal vascularity in the left lateral wall of the bladder projecting into the lumen. A 1.9‐cm bladder calculus was seen in the bladder (Fig. 3).

He underwent vesicolitholapaxy and transurethral resection of bladder tumor (TURBT). The stone was seen embedded deeply within the tumor and required resection to free the stone for litholapaxy. A large solid exophytic tumor with a whitish core and evidence of increased vascularity, involving the left lateral wall of the bladder and the trigone, was resected almost fully. He made a slow recovery after the initial surgery complicated by culture positive urinary tract infection.

**Figure 4:**
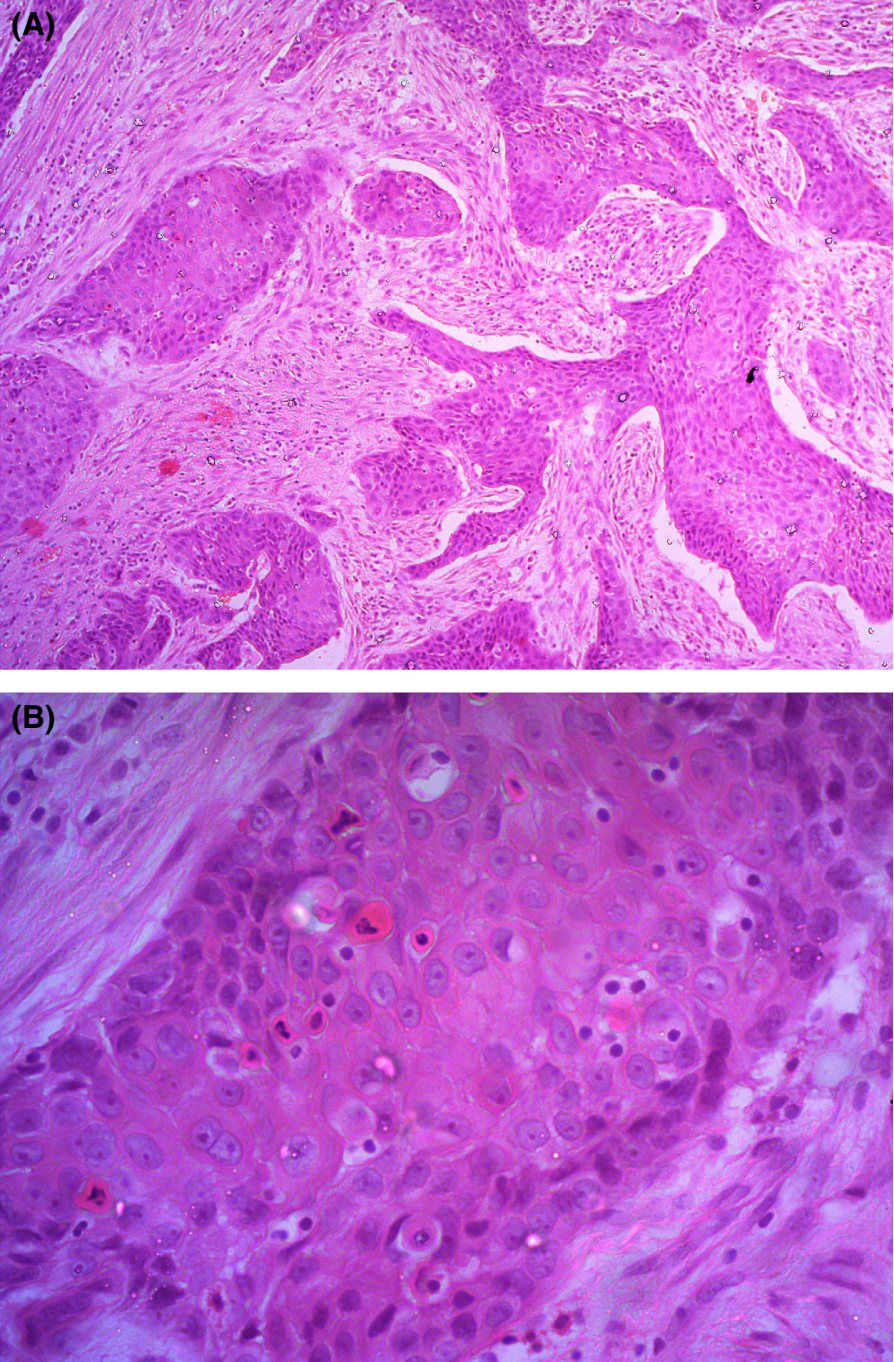
(A and B) H & E staining viewed under x 10 and x 40 (respectively) showing a well differentiated squamous cell carcinoma with invasion of lamina propria and muscularis propria and absent lymphovascular invasion.

Histopathology revealed a well‐differentiated squamous cell carcinoma with invasion of lamina propria and muscularis propria and absent lymphovascular invasion compatible with a pathological tumor stage of pT2 (Fig. 4).

**Figure 5:**
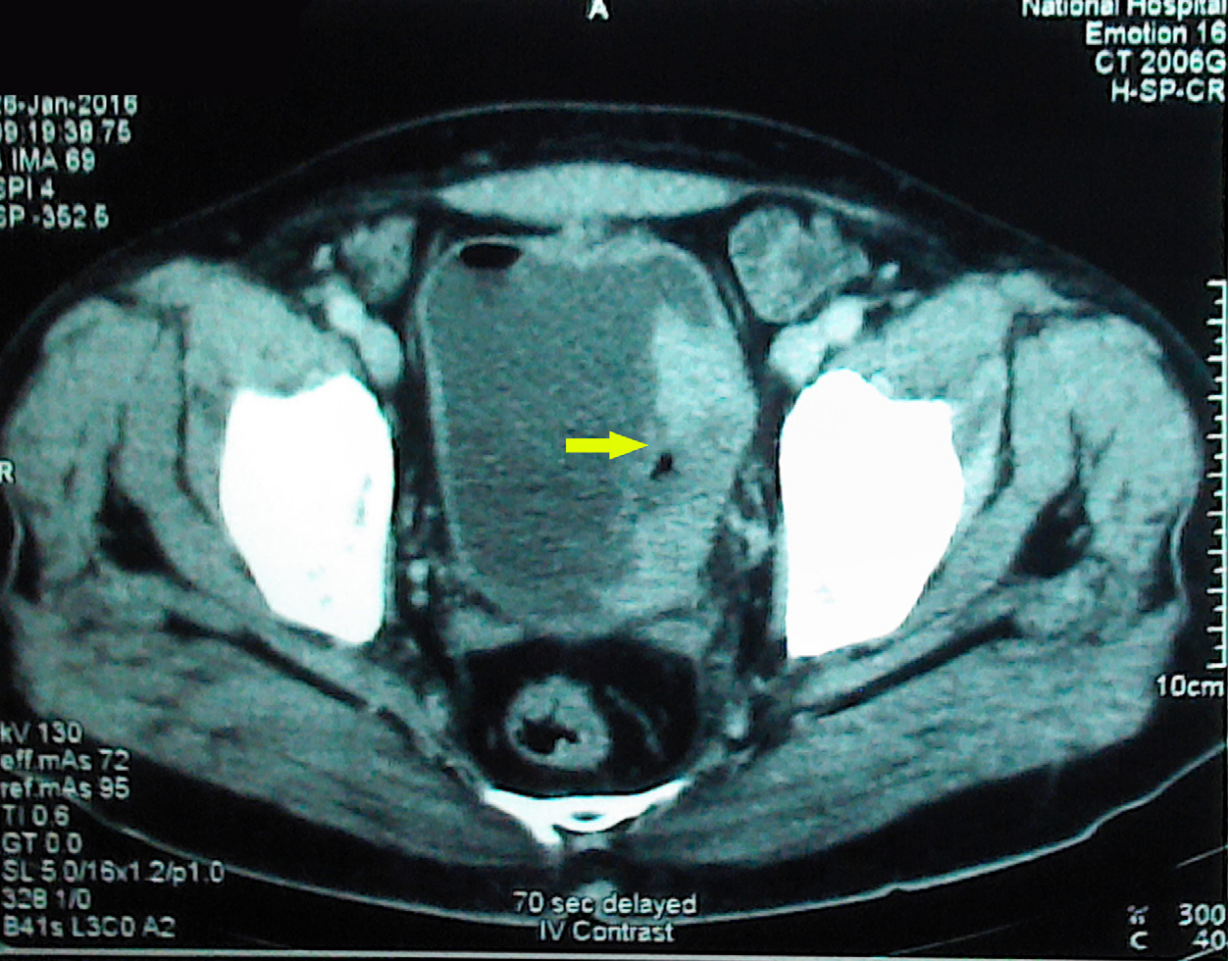
CT scan of the abdomen and pelvis showing a homogenously enhancing mass lesion in the left inferior lateral wall of the bladder measuring 5 × 4 × 4 cm, involving the left vesicoureteric junction.

Staging CECT (contrast‐enhanced computed tomography) abdomen and pelvis (Fig. 5) performed 26 days after the initial TURBT showed a considerably large homogenously enhancing mass lesion in the left inferior lateral wall of the bladder measuring 5 × 4 × 4 cm, involving the left vesicoureteric junction. There was a perivesical 6‐mm solitary lymph node and fat stranding, raising the suspicion of perivesical and prostatic spread. These findings suggested an aggressive tumor, considering the gap of 26 days between initial near total transurethral resection and CECT.

Staging Technetium 99 m‐MDP (methyl diphosphonate) bone scan was negative for bone metastasis. He was offered radical cystoprostatectomy and ileal conduit reconstruction which was abandoned due to the extensive nodal disease in the pelvis. Pelvic nodal sampling detected presence of metastases in four out of 10 nodes sampled. The patient succumbed to the disease within 9 months following diagnosis.

## Discussion

Squamous cell carcinoma of the bladder (SCCB) is a rare cause of bladder cancer, accounting for 2.7% of bladder cancers in the developed world 1. In areas where schistosomiasis is endemic, it is the commonest cause of bladder cancer which accounts to around 59% of bladder cancers 3. SCCB can be subclassified as bilharzial and nonbilharzial depending on the etiology due to *Schistosomiasis hematobium* 4, 5. Nonbilharzial squamous cell carcinoma is associated with conditions causing chronic bladder irritation which are bladder stones, recurrent urinary tract infections, chronic bladder outlet obstruction, indwelling catheters, cyclophosphamide exposure and even intravesical Bacillus Calmette‐Guerin (BCG) which result in subsequent metaplasia and malignant transformation 5, 6.

Squamous cell carcinoma of the bladder usually presents at an advanced stage and has a significantly high mortality than urothelial bladder cancers. Approximately 56% of all SCCB were graded as American Joint Committee on Cancer (AJCC) stage T3 or T4 at presentation. Furthermore, in a large series of 1422 patients with nonbilharzial SCCB, about 85% were muscle invasive at diagnosis 7. Surgery is the mainstay of treatment of squamous cell carcinoma of the bladder; however, there is an associated high recurrence and poor prognosis following surgery 8.

Large vesical calculi were mentioned as those weighing more than 100 g (approximately diameter of 4 cm) 9. In another study, large bladder stone was taken as diameter of more than 3 cm 10. Few studies have evaluated the association of bladder stones in relation to bladder cancer. A case–control study has shown that a twofold increase in bladder cancer risk was observed with a history of bladder stones, irrespective of a history of urinary tract infections 11. In another case–control study, a multivariate analysis revealed that in patients with newly diagnosed bladder cancer, the odds ratio of having been diagnosed with a bladder calculus was 3.42 (95% CI  =  2.48–4.72) when compared with controls. In another prospective cohort study of kidney or ureteral stones, the standardized incidence ratio for bladder cancer was 1.4 (95% CI (confidence interval) 1.3–1.6, *n* = 319) 12. However, there are no large scale studies that have looked at the percentage of squamous cell carcinoma diagnosed with a concomitant bladder calculus. This is probably because of the rarity of the disease. Furthermore, the occurrence of squamous cell carcinoma of the bladder by histological analysis, subsequent to a stone, is yet to be studied. Thus, it may be useful to conduct future prospective studies to study the occurrence of carcinoma‐in‐situ or dysplasia in patients with large bladder stones.

The reported patient had a history of large vesical calculus 3 years back, and at that time, there was no cystoscopic evidence of mucosal abnormality suggestive of a malignancy. Furthermore, he was clinically asymptomatic for almost 3 years following which he developed lower urinary tract symptoms with hematuria. Unfortunately he had aggressive advanced disease with extensive pelvic nodal disease at the time of diagnosis. Although the exact reason for the aggressiveness of the tumor is not clear, the extensive pelvic metastasis could have contributed to the aggressive behavior of the tumor.

Therefore in patients with large bladder stones, we suggest random biopsies to detect presence of dysplasia or malignancy and a follow‐up cystoscopy to exclude a tumor. Furthermore, cystoscopic surveillance may be useful to look for asymptomatic recurrent bladder stones which will further predispose to a malignancy. However, the cost effectiveness of the suggested practice can only be confirmed by further studies. As SCCB is usually advanced at presentation, early detection is key for better prognosis.

## Conclusion

We present a case of primary SCCB with a past history of large bladder stone and stone recurrence. The patient was found to have an advanced SCCB at presentation, and the disease was rapidly progressive resulting in mortality within 9 months following diagnosis. Therefore, in patients with large bladder stones, which suggests long‐standing chronic irritation of the bladder, we suggest random biopsies to detect the presence of dysplasia or malignancy and a follow‐up flexible cystoscopy. This is to detect the tumor early which is key for better prognosis.

## Consent

Informed written consent was obtained from the patient prior to collecting information.

## Authorship

DMHF, UJ, and KBH: contributed to collection of information and writing of the manuscript. Authors MVCS; SASG: contributed to writing and final approval of the manuscript.

## Conflict of Interest

Authors declared that there are no conflict of interests.

## References

[1] Rogers, C. G. , G. S. Palapattu , S. F. Shariat , P. I. Karakiewicz , P. J. Bastian , Y. Lotan , et al. 2006 Clinical outcomes following radical cystectomy for primary nontransitional cell carcinoma of the bladder compared to transitional cell carcinoma of the bladder. J. Urol. 175:2048–2053; discussion 2053. 16697800 10.1016/S0022-5347(06)00317-X

[2] Goonewardena, S. , W. De Silva , and M. De Silva . 2004 Bladder cancer in Sri Lanka: experience from a tertiary referral center. Int. J. Urol. 11:969–972. 15509199 10.1111/j.1442-2042.2004.00930.x

[3] Ghoneim, M. A. , M. M. el‐Mekresh , M. A. el‐Baz , I. A. el‐Attar , and A. Ashamallah . 1997 Radical cystectomy for carcinoma of the bladder: critical evaluation of the results in 1,026 cases. J. Urol. 158:393–399. 9224310

[4] El‐Bolkainy, M. , N. Mokhtar , M. Ghoneim , and M. Hussein . 1981 The impact of schistosomiasis on the pathology of bladder carcinoma. Cancer 48:2643–2648. 7306921 10.1002/1097-0142(19811215)48:12<2643::aid-cncr2820481216>3.0.co;2-c

[5] Kassouf, W. , P. E. Spiess , A. Siefker‐Radtke , D. Swanson , H. B. Grossman , A. M. Kamat , et al. 2007 Outcome and patterns of recurrence of nonbilharzial pure squamous cell carcinoma of the bladder. Cancer 110:764–769. 17614317 10.1002/cncr.22853

[6] Dahm, P. , and J. E. Gschwend . 2003 Malignant non‐urothelial neoplasms of the urinary bladder: a review. Eur. Urol. 44:672–681. 14644119 10.1016/s0302-2838(03)00416-0

[7] Scosyrev, E. , J. Yao , and E. Messing . 2009 Urothelial carcinoma versus squamous cell carcinoma of bladder: is survival different with stage adjustment? Urology 73:822–827. 19193403 10.1016/j.urology.2008.11.042

[8] Chalasani, V. , J. L. Chin , and J. I. Izawa . 2009 Histologic variants of urothelial bladder cancer and nonurothelial histology in bladder cancer. Can. Urol. Assoc. J. 3(6 Suppl 4):S193–S198. 20019984 10.5489/cuaj.1195 PMC2792446

[9] Tahtalı, İ. N. , and T. Karataş . 2014 Giant bladder stone: a case report and review of the literature. Turk. J. Urol. 40:189–191. 26328176 10.5152/tud.2014.02603 PMC4548388

[10] Toktas, G. , V. Sacak , E. Erkan , R. Kocaaslan , M. Demiray , E. Unluer , et al. 2013 Novel technique of cytolithotripsy for large bladder stones. Asian J. Endosc. Surg. 6:245–248. 23879422 10.1111/ases.12029

[11] Kantor, A. F. , P. Hartge , R. N. Hoover , A. S. Narayana , J. W. Sullivan , and J. F. Jr Fraumeni . 1984 Urinary tract infection and risk of bladder cancer. Am. J. Epidemiol. 119:510–515. 6711540 10.1093/oxfordjournals.aje.a113768

[12] Michaud, D. S. 2007 Chronic inflammation and bladder cancer. In Urologic oncology: Seminars and original investigations 25(3): pp. 260–268. Elsevier. https://doi.org/10.1016/j.urolonc.2006.10.002. 10.1016/j.urolonc.2006.10.002 17483025

